# Author Correction: Recurrent acquisition of cytosine methyltransferases into eukaryotic retrotransposons

**DOI:** 10.1038/s41467-018-04260-2

**Published:** 2018-05-01

**Authors:** Alex de Mendoza, Amandine Bonnet, Dulce B. Vargas-Landin, Nanjing Ji, Hongfei Li, Feng Yang, Ling Li, Koichi Hori, Jahnvi Pflueger, Sam Buckberry, Hiroyuki Ohta, Nedeljka Rosic, Pascale Lesage, Senjie Lin, Ryan Lister

**Affiliations:** 1 Australian Research Council Centre of Excellence in Plant Energy Biology, School of Molecular Sciences, The University of Western Australia, Perth, WA 6009 Australia; 2 Harry Perkins Institute of Medical Research, Perth, WA 6009 Australia; 3 Université Paris Diderot, Sorbonne Paris Cité, INSERM U944, CNRS UMR 7212, Institut Universitaire d’Hématologie, Hôpital St. Louis, 75010 Paris, France; 4 State Key Laboratory of Marine Environmental Science and College of Ocean and Earth Sciences, Xiamen University, 361102 Xiamen, China; 5 School of Life Science and Technology, Tokyo Institute of Technology, Yokohama City, Kanagawa 226-8501 Japan; 6 School of Health and Human Sciences, Southern Cross University, Gold Coast, QLD 4072 Australia; 7 School of Biological Sciences, The University of Queensland, St. Lucia, QLD 4225 Australia; 8 Institute of Systems Genomics, University of Connecticut, Storrs, CT 06269 USA; 9 Department of Marine Sciences, University of Connecticut, Groton, CT 06340 USA

Correction to: *Nature Communications*; 10.1038/s41467-018-03724-9 published online 09 April 2018

The original version of this Article contained an error in the spelling of the author Hongfei Li, which was incorrectly given as Fei Hong. This has now been corrected in both the PDF and HTML versions of the Article.

