# Supplementary material for: Recurrent acquisition of cytosine methyltransferases into eukaryotic retrotransposons
Source: Nat Commun. 2018 Apr 9;9:1341. doi: 10.1038/s41467-018-03724-9 (PMC5890265; doi:10.1038/s41467-018-03724-9)
Supplement: Supplementary file 1 — Supplementary Information [file 41467_2018_3724_MOESM1_ESM.pdf]

Recurrent acquisition of cytosine methyltransferases into eukaryotic retrotransposons

de Mendoza et al.

Supplementary Figures

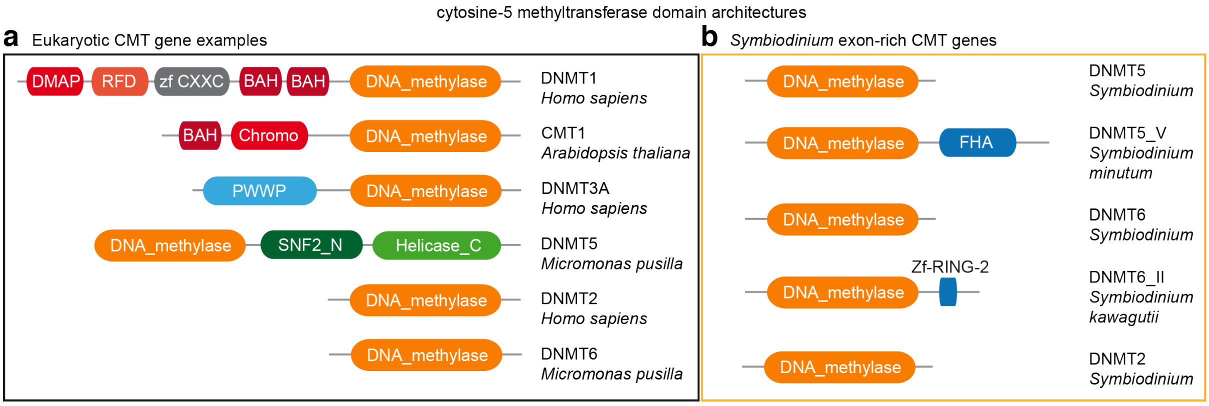

Supplementary Figure 1. *Symbiodinium* multi-exonic cytosine methyltransferases have simplified domain architectures compared to their eukaryotic counterparts. (**a**) Domain architecture configuration of representative eukaryotic DNMTs. (**b**) Domain architectures of the DNMTs encoded in multi-exonic genes of *Symbiodinium* genomes. Unlike other eukaryotic DNMTs, *Symbiodinium* DNMTs lack typical domains such as PWWP or BAH chromatin interaction domains, that are found in animal or plant Dnmt1 and Dnmt3 members. Similarly, the C-terminal SNF2 domain typical of the Dnmt5 family is absent in *Symbiodinium* orthologs[11]. A unique exception is a *S. minutum* paralog of DNMT5, harbouring a FHA (Forkhead-associated) domain, which so far has not been associated to any other DNMT in eukaryotes. FHA domain is a phosphopeptide recognition domain, which would suggest a post-translational control of this specific paralog by kinases. In *S. kawagutii* a single paralog of DNMT6 encodes an extra Zf-RING-2 domain, related to the ubiquitin signaling. All the protein domains are defined according to PFAM A database models.

a

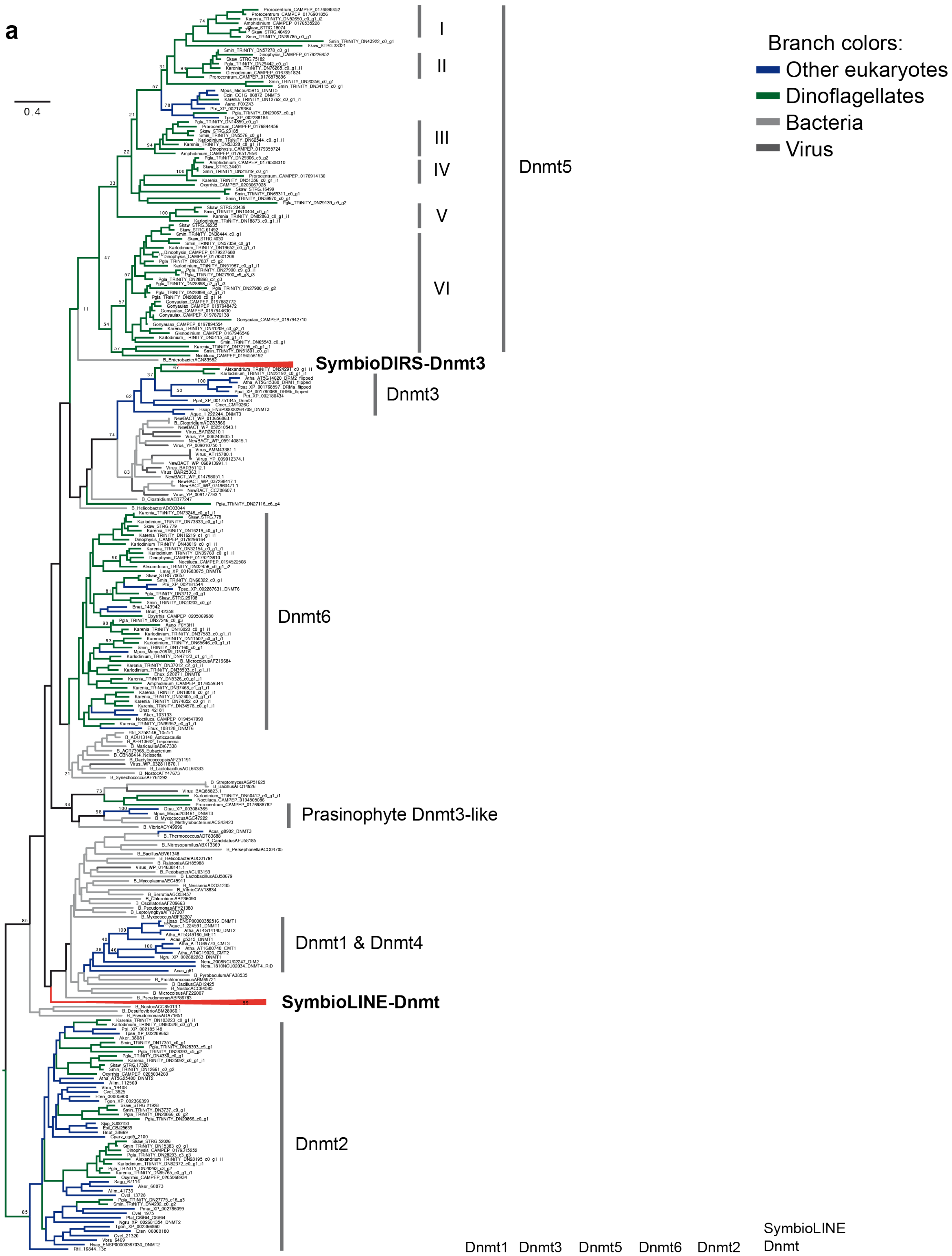

b

		Dnmt1	Dnmt3	Dnmt5	Dnmt6	Dnmt2	SymbioLINE Dnmt
Gymnodiniphycidae	*Symbiodinium kawagutii*	-	401	11	4	3	738
	Symbiodinium minutum	-	85	14	3	5	90
	Polarella glacialis	-	-	8	2	5	-
	Amphidinium carterae	-	-	3	1	-	-
	Karenia brevis	-	-	8	14	3	-
	Karlodinium micrum	-	1	5	7	2	-
Peridiniphycidae	*Alexandrium fundyense*	-	1	-	1	1	-
	Gonyaulax spinifera	-	-	-	6	-	-
	Glenodinium foliaceum	-	-	2	-	-	-
Dinophysiales	*Dinophysis acuminata*	-	-	4	2	1	-
Prorocentrales	*Prorocentrum minimum*	-	-	5	-	-	-
Noctilucales	*Noctiluca scintillans*	-	-	1	2	-	-
Oxyrrhis	*Oxyrrhis marina*	-	-	1	1	2	-

Supplementary Figure 2. DNMT repertoire is conserved across dinoflagellates. (**a**) Maximum likelihood phylogenetic tree of DNMT proteins from dinoflagellate transcriptomes. Nodal supports are 100 replicates of parametric bootstrap. The clade of DNMT5 shows several well-supported subfamilies conserved across different dinoflagellate species, which indicates ancient duplications conserved across the group. (**b**) Distribution of DNMT families in 13 dinoflagellate species. Highlighted in red the DNMT families found in retrotransposons. Dnmt3 orthologs are found in only two species, but not associated to retrotranscriptase domains.

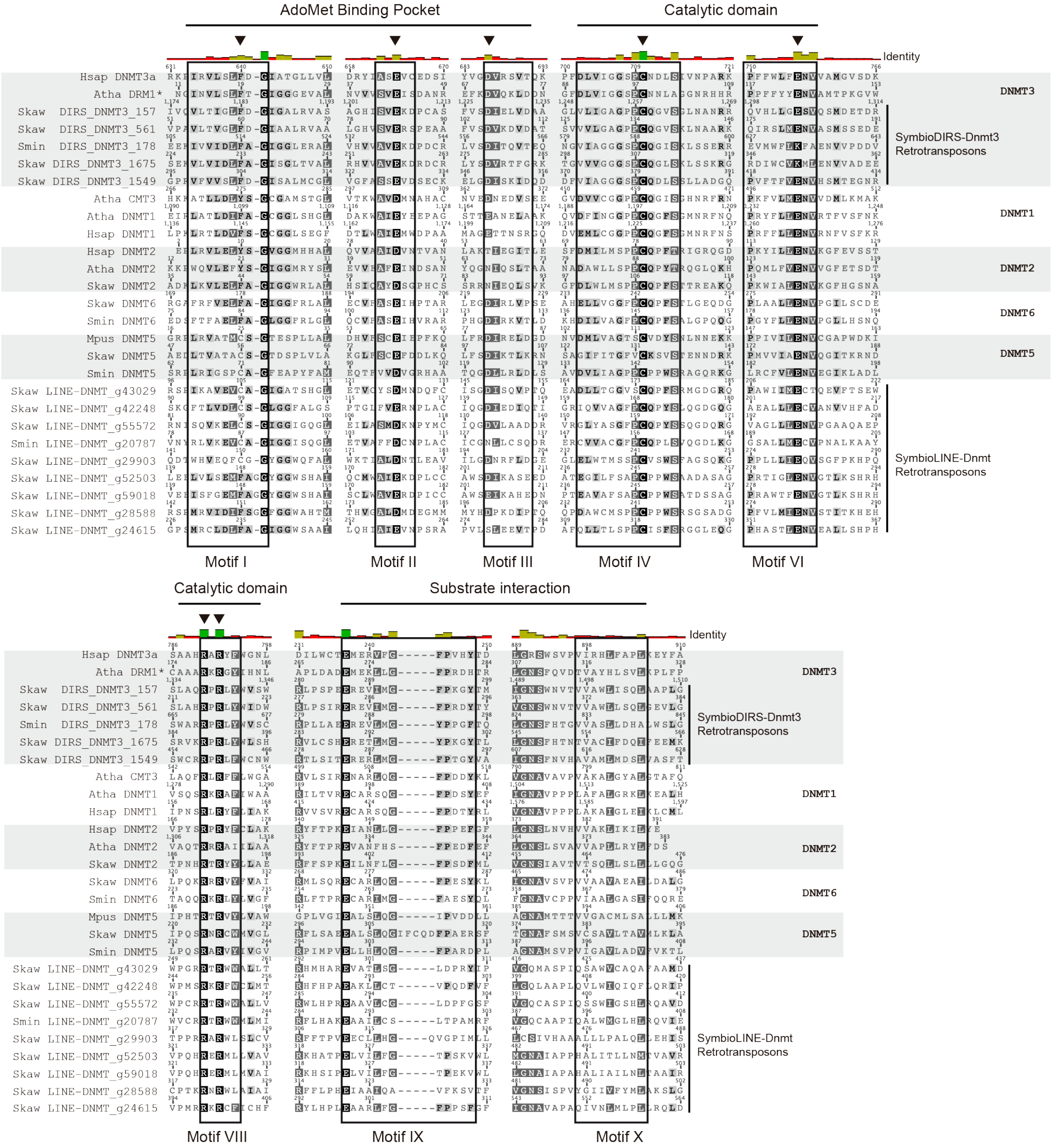

Supplementary Figure 3. Key amino acid positions are conserved in *Symbiodinium* retrotransposon associated DNMTs. Multiple sequence alignment showing representative sequences of the distinct DNMTs encoded in *Symbiodinium* compared to representatives of functionally characterized DNMT classes. The diagnostic functional motifs [1,2] are well conserved in all types, including the retrotransposons. The key functional amino acids are highlighted with a black triangle. Abbreviations: Hsap (*Homo sapiens*), Atha (*Arabidopsis thaliana*), Mpus (*Micromonas pusilla*), Skaw (*S. kawagutii*), Smin (*S. minutum*).

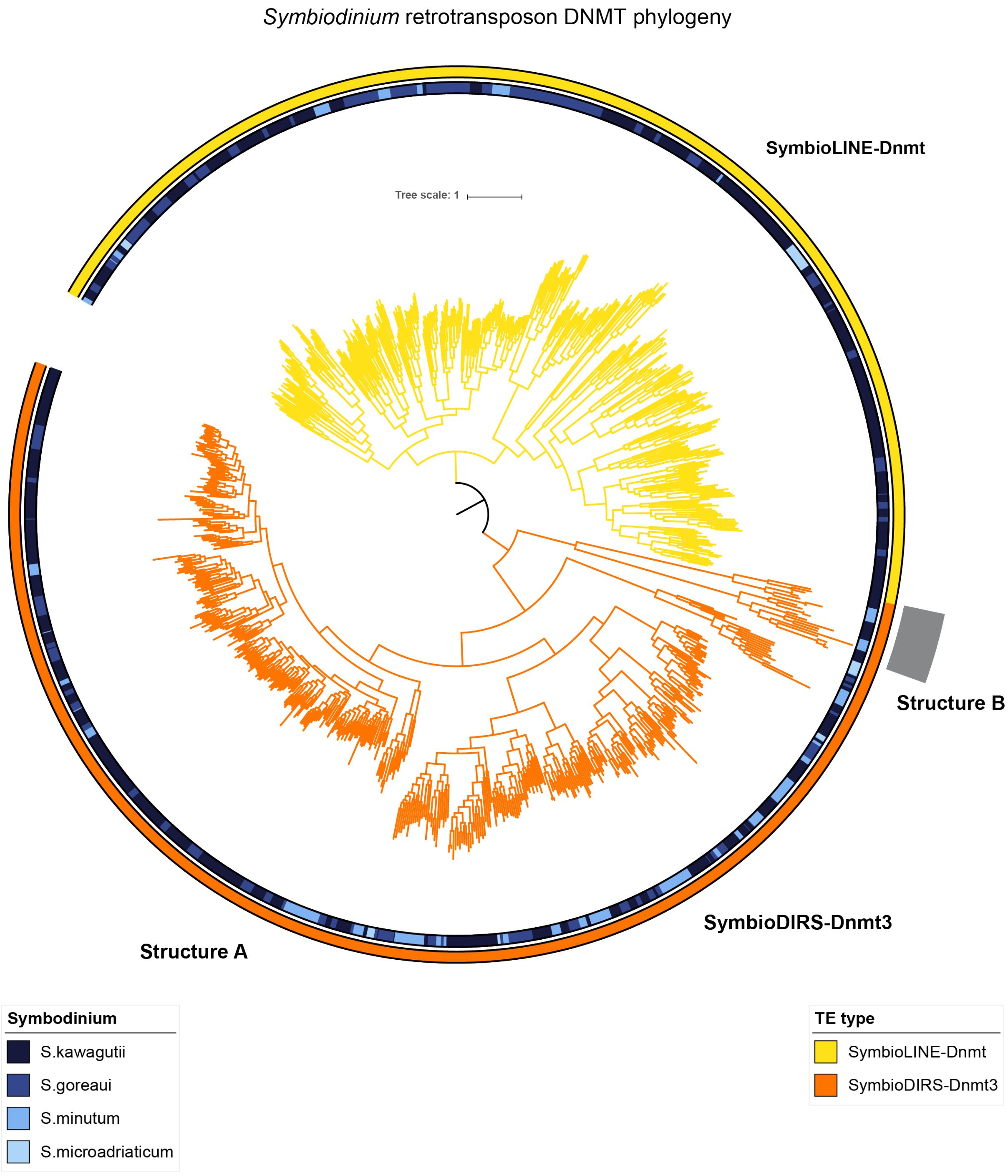

Supplementary Figure 4. Retrotransposon DNMT phylogeny in *Symbiodinium* genomes. Maximum likelihood phylogenetic tree based on the DNMT domains encoded in SymbioLINE-Dnmt and SymbioDIRS-Dnmt3 retrotransposons. The internal circle indicates species affiliation of each sequence and the outer circle indicates the type of retrotransposon were each DNMT domain is found. In grey are highlighted the DNMT sequences that belong

to SymbioDIRS-Dnmt3 structure B, while the rest belong to SymbioDIRS-Dnmt3 structure A.

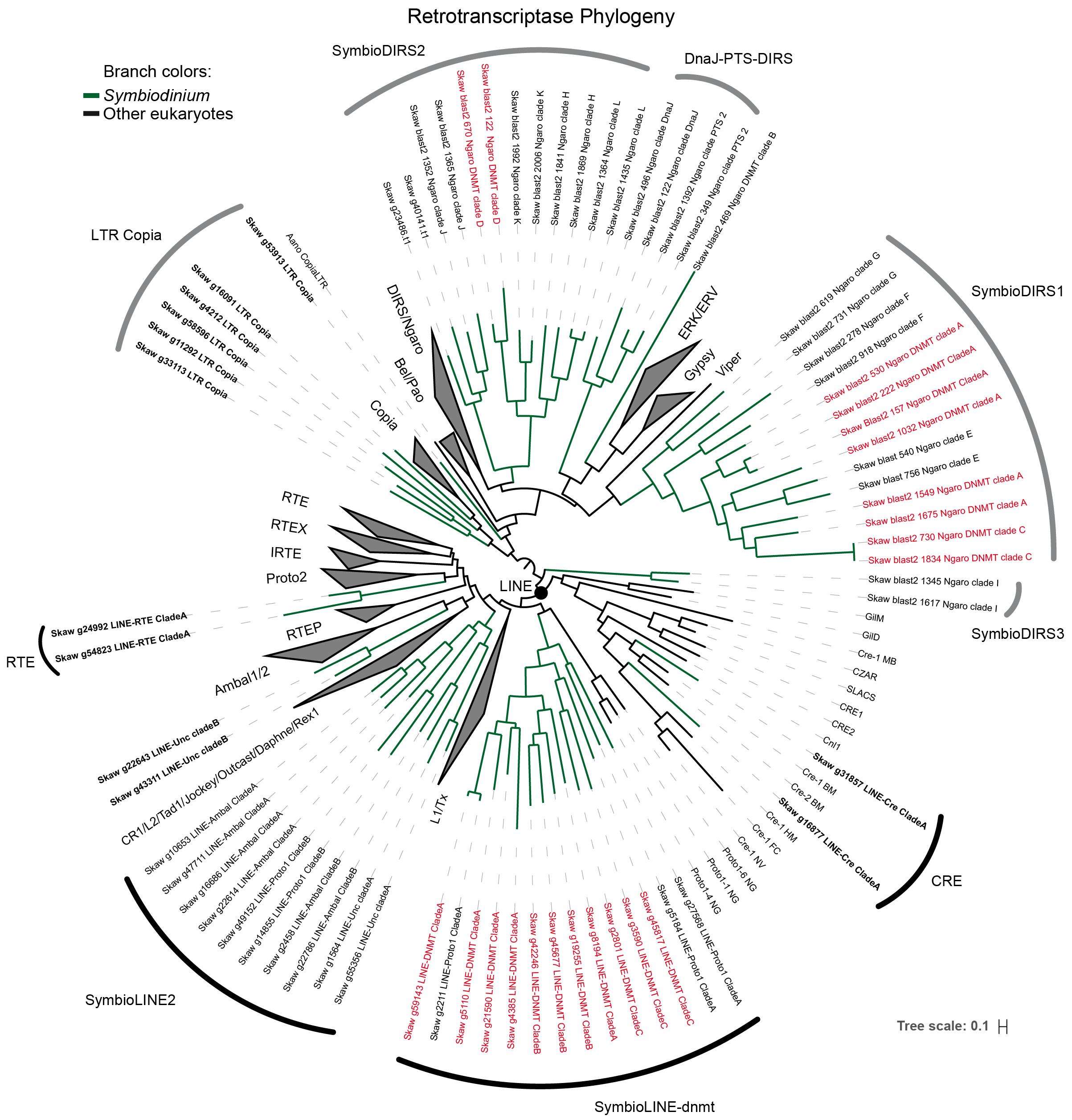

Supplementary Figure 5. *Symbiodinium* genomes encode several families of retrotransposons. Maximum likelihood phylogenetic tree of the reverse transcriptase domain, including representative sequences of *Symbiodinium* (selected to capture the major clades in Figure 2a,b) and a subset of sequences from RepBase. Established clades without dinoflagellate sequences are collapsed. SymbioLINE-Dnmt and SymbioDIRS-Dnmt3 are highlighted in red. *Symbiodinium* sequences are quite divergent and evolve fast, which diminishes nodal support in deeper branches. LINE elements mostly cluster in *Symbiodinium*-specific clades (CRE and RTE related sequences are the exceptions). DIRS elements appear

in different poorly supported clades, which reflect the divergence of this class of retrotransposons. At least 4 major clades are observed, two of them with SymbioDIRS-Dnmt3 representatives. SymbioDIRS1 clade includes the sequences with SymbioDIRS-Dnmt3 structure A (plus others without DNMT), and SymbioDIRS2 includes the sequences with SymbioDIRS-Dnmt3 structure B (also clustering with other DIRS elements lacking a DNMT). SymbioDIRS3 clade lacks DNMT and has a reverse transcriptase more closely related to LINEs, despite having a structure similar to DIRS retrotransposons. Finally, DnaJ-PTS-DIRS is a small clade that has incorporated DnaJ (chaperone) or PTS (RNA binding) domains into their *Pol* ORF, showing how other domains than DNMT have been acquired by *Symbiodinium* DIRS retrotransposons, although fewer copies are found in the genome.

a

b

c

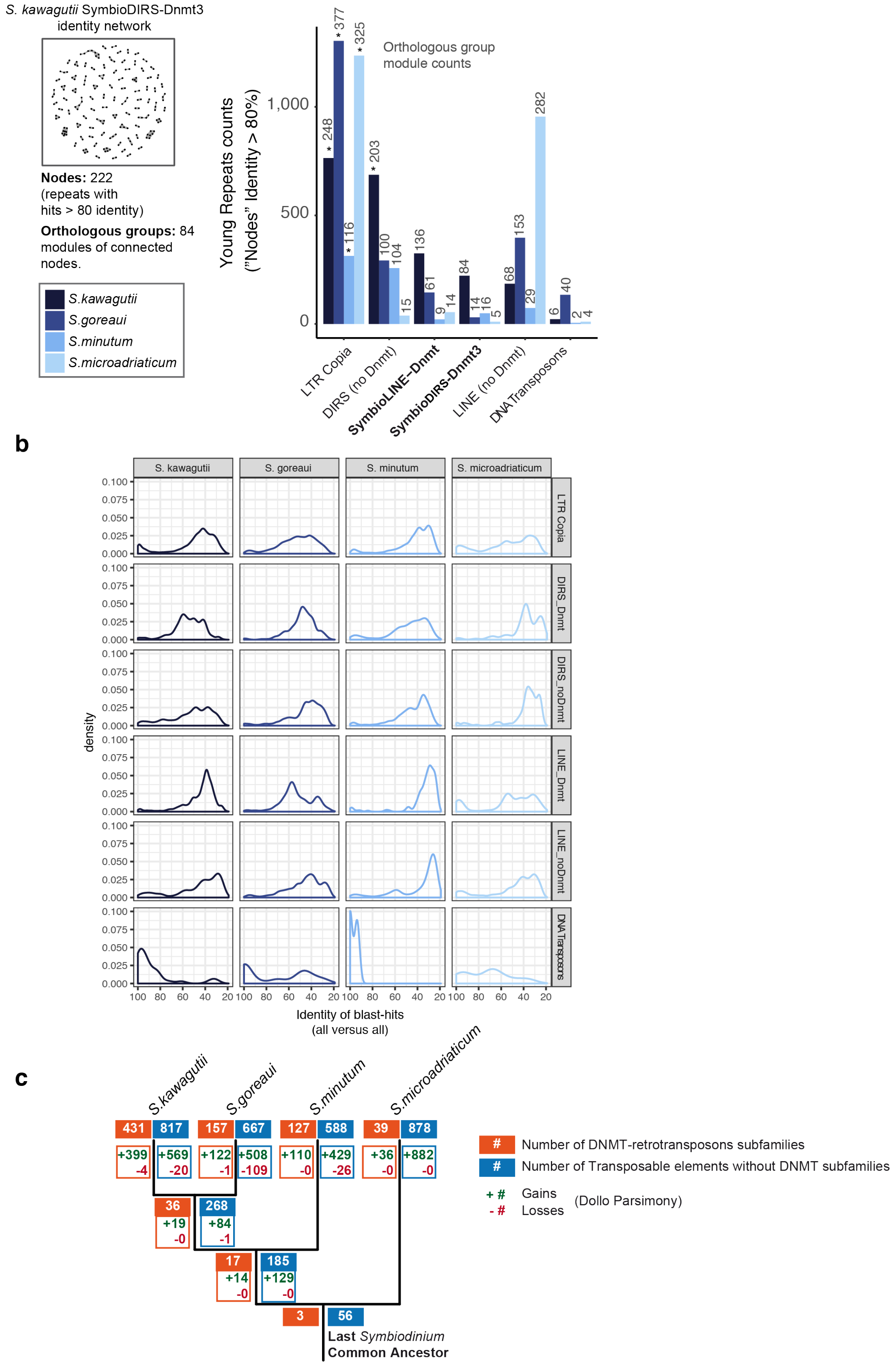

S. kawagutii SymbioDIRS-Dnmt3 identity network
Nodes: 222 (repeats with hits > 80 identity)
Orthologous groups: 84 modules of connected nodes.
S.kawagutii
S.goreaui
S.minutum
S.microadriaticum
Young Repeats counts ("Nodes" Identity > 80%)
Orthologous group module counts
1,000
500
0
* 248
* 377
* 116
* 325
* 203
100
104
15
136
61
9
14
84
14
16
5
68
153
29
282
6
40
2
4
LTR Copia
DIRS (no Dnmt)
SymbioLINE-Dnmt
SymbioDIRS-Dnmt3
LINE (no Dnmt)
DNA Transposons
S. kawagutii
S. goreaui
S. minutum
S. microadriaticum
LTR Copia
DIRS_Dnmt
DIRS_noDnmt
LINE_Dnmt
LINE_noDnmt
DNA Transposons
density
0.100
0.075
0.050
0.025
0.000
100 80 60 40 20
Identity of blast-hits (all versus all)
S.kawagutii
S.goreaui
S.minutum
S.microadriaticum
431
817
+399
-4
+569
-20
157
667
+122
-1
+508
-109
127
588
+110
-0
+429
-26
39
878
+36
-0
+882
-0
36
268
+19
-0
+84
-1
17
185
+14
-0
+129
-0
3
56
Last Symbiodinium Common Ancestor
Number of DNMT-retrotransposons subfamilies
Number of Transposable elements without DNMT subfamilies
+ # Gains
- # Losses
(Dollo Parsimony)

Supplementary Figure 6. Different types of transposable elements are active in *Symbiodinium* *genomes.* (**a**) Each bar represents the total number of young transposable element copies in each *Symbiodinium* species (as having > 80% amino acid identity to other transposons in the same genome). Asterisk indicates which types are enriched when compared to the total number of copies of Fig. 2c (one sided fisher exact test $p< 0.01$). In grey is the number of "modules" in each similarity network, defining groups of young transposable elements that are connected by Identity values >80% as exemplified by the SymbioDIRS-Dnmt3 *S. kawagutii* network on the left, where 222 young copies can be summarised into 84 orthologous groups. (**b**) Distribution of pairwise amino acid sequence identities between all transposable element types belonging to each genome as obtained from blastp all versus all searches. Self-hits (a query against itself) have been removed. All retrotransposon types show most copies having diverged substantially from each other, but all retrotransposon types present a small fraction of young copies (similar to other transposons encoded in the genome). This is consistent with several divergent retrotransposon families being active at the same time in these genomes. In contrast, most DNA transposons are younger, as most copies resemble other copies in the genome, indicating that few families are actively transposing. (**c**) Phylogenetic reconstruction of transposable element subfamily evolution in *Symbiodinium* genomes. Orthologous groups were defined as having >60% amino acid identity to other transposons and evolutionary patterns and ancestral node reconstructions were obtained using Dollo parsimony. In orange evolution of DNMT containing retrotransposons (both SymbioDIRS-Dnmt3 and SymbioLINE-Dnmt) and in blue the rest of transposable elements, including gains and losses for each type.

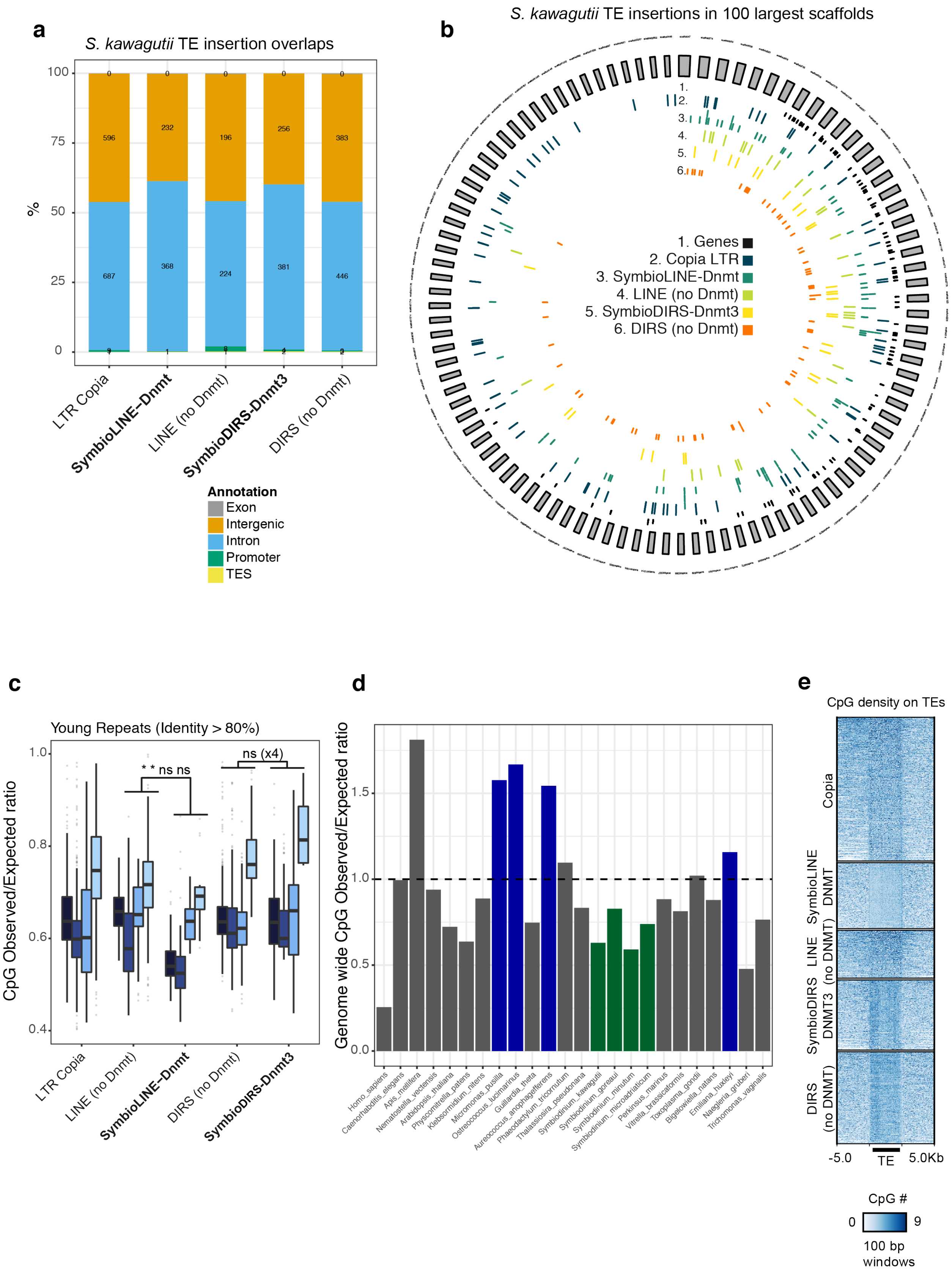

a
S. kawagutii TE insertion overlaps
%
100
75
50
25
0
596
232
196
256
383
687
368
224
381
446
LTR Copia
SymbioLINE-Dnmt
LINE (no Dnmt)
SymbioDIRS-Dnmt3
DIRS (no Dnmt)
Annotation
Exon
Intergenic
Intron
Promoter
TES
b
S. kawagutii TE insertions in 100 largest scaffolds
1. Genes
2. Copia LTR
3. SymbioLINE-Dnmt
4. LINE (no Dnmt)
5. SymbioDIRS-Dnmt3
6. DIRS (no Dnmt)
c
Young Repeats (Identity > 80%)
CpG Observed/Expected ratio
1.0
0.8
0.6
0.4
* * ns ns
ns (x4)
LTR Copia
LINE (no Dnmt)
SymbioLINE-Dnmt
DIRS (no Dnmt)
SymbioDIRS-Dnmt3
d
Genome wide CpG Observed/Expected ratio
1.5
1.0
0.5
0.0
Homo_sapiens
Caenorhabditis_elegans
Apis_mellifera
Nematostella_vectensis
Arabidopsis_thaliana
Physcomitrella_patens
Klebsormidium_nitens
Micromonas_pusilla
Ostreococcus_lucimarinus
Guillardia_theta
Aureococcus_anophagefferens
Phaeodactylum_tricornutum
Thalassiosira_pseudonana
Symbiodinium_kawagutii
Symbiodinium_goreaui
Symbiodinium_minutum
Symbiodinium_microadriaticum
Perkinsus_marinus
Vitrella_brassicaformis
Toxoplasma_gondii
Bigelowiella_natans
Emiliana_huxleyi
Naegleria_gruberi
Trichomonas_vaginalis
e
CpG density on TEs
Copia
SymbioLINE DNMT
LINE (no DNMT)
SymbioDIRS DNMT3
DIRS (no DNMT)
-5.0
TE
5.0Kb
CpG #
0
9
100 bp windows

Supplementary Figure 7. ***Symbiodinium*** **retrotransposon insertion patterns and genome-wide CG dinucleotide depletion.** (**a**) Retrotransposon insertions in S. kawagutii genome classified as overlapping genomic features obtained from the Stringtie annotation. Color code as in legend below. (**b**) Retrotransposon insertion distribution across the largest 100 scaffolds of *S. kawagutii* (1st= 1.9 Mb, 100th = 0.8 Mb). Each concentric circle shows a track for each feature type location as specified by the legend at the center of the plot. (**c**) Comparison of the CG observed/expected ratio across young copies of all retrotransposon types. SymbioLINE-DNMTs have fewer CGs than the rest (asterisks represent Wilcoxon one sided rank-sum test $p<0.01$) of retrotransposons in *S. kawagutii* and *S. goreaui*, while SymbioDIRS-Dnmt do not show a significant difference when compared to DIRS elements without DNMT (ns represent $p>0.01$). Boxplot centre lines are medians, box limits are quartiles 1 (Q1) and 3 (Q3), whiskers are 1.5 x interquartile range (IQR) and points are outliers. (**d**) Genomic CG dinucleotide Observed versus Expected ratios of diverse eukaryotes. Blue represents the species that have periodic DNA methylation in nucleosome linker sequences mediated by DNMT5 enzymes[3]. Green represents *Symbiodinium* species. *Symbiodinium* depletion of CG dinucleotides indicates that mutagenic effects of methylated cytosines is a force shaping the genomic base composition of all *Symbiodinium* species. (**e**) Heatmap showing CG dinucleotide density on transposable elements. SymbioLINE-Dnmt show a reduced CG dinucleotide density, as observed in Figure 2d.

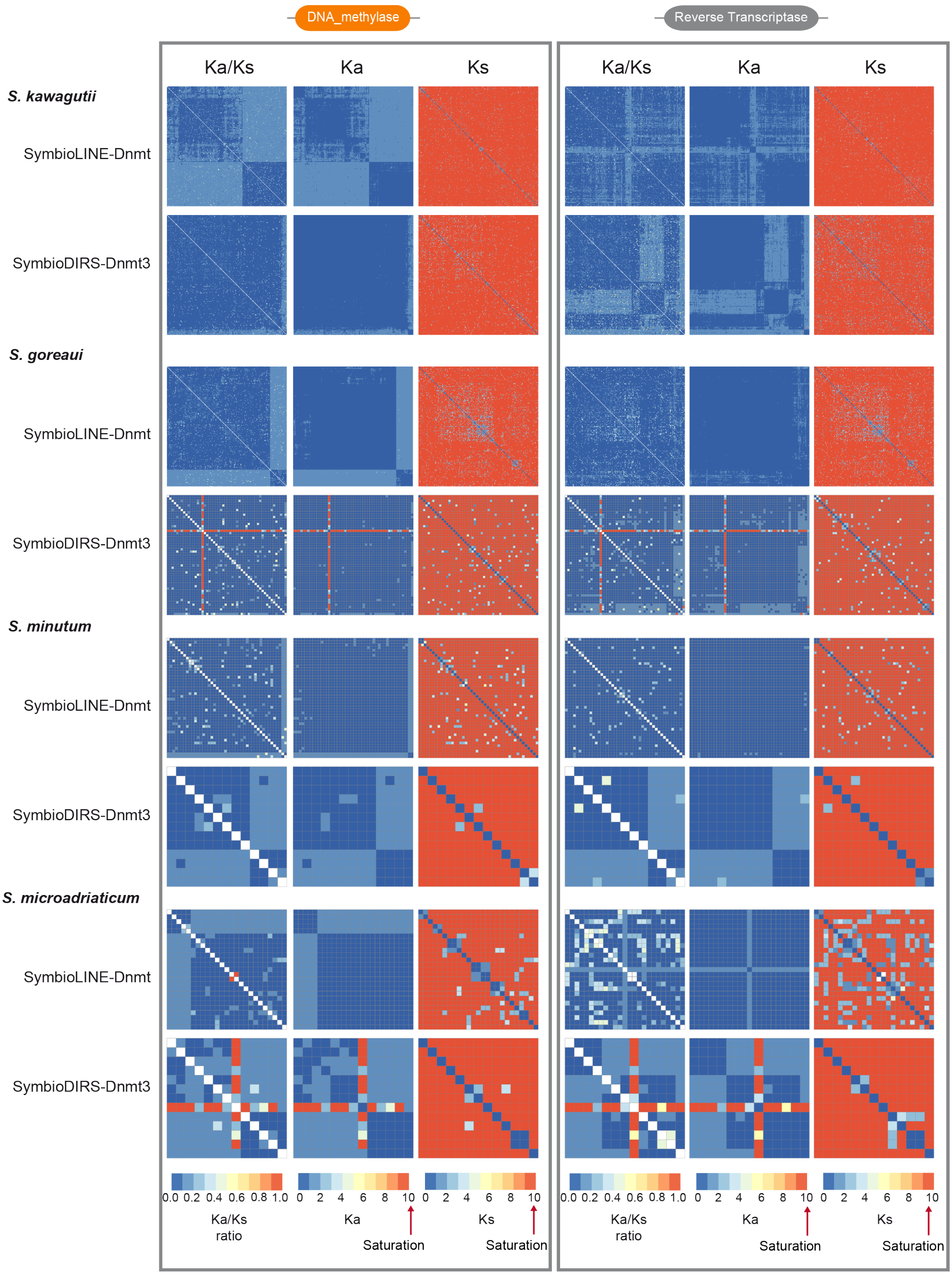

Supplementary Figure 8. Retrotransposon DNMT show evidence of purifying selection. Heatmaps showing all versus all pair-wise codon alignments between DNMT and reverse Transcriptase domains belonging to SymbioDIRS-Dnmt3 and SymbioLINE-Dnmt from 4

Symbiodinium species. Ordering of the sequences from DNMT and reverse transcriptase domains belonging to the same retrotransposons is matched across heatmaps. Ks and Ka values close to 10 represent saturated substitution rates. Self-comparisons of Ka/Ks ratios are shown in white across the diagonal. Neutral selection is estimated as Ka/Ks ~ 1.

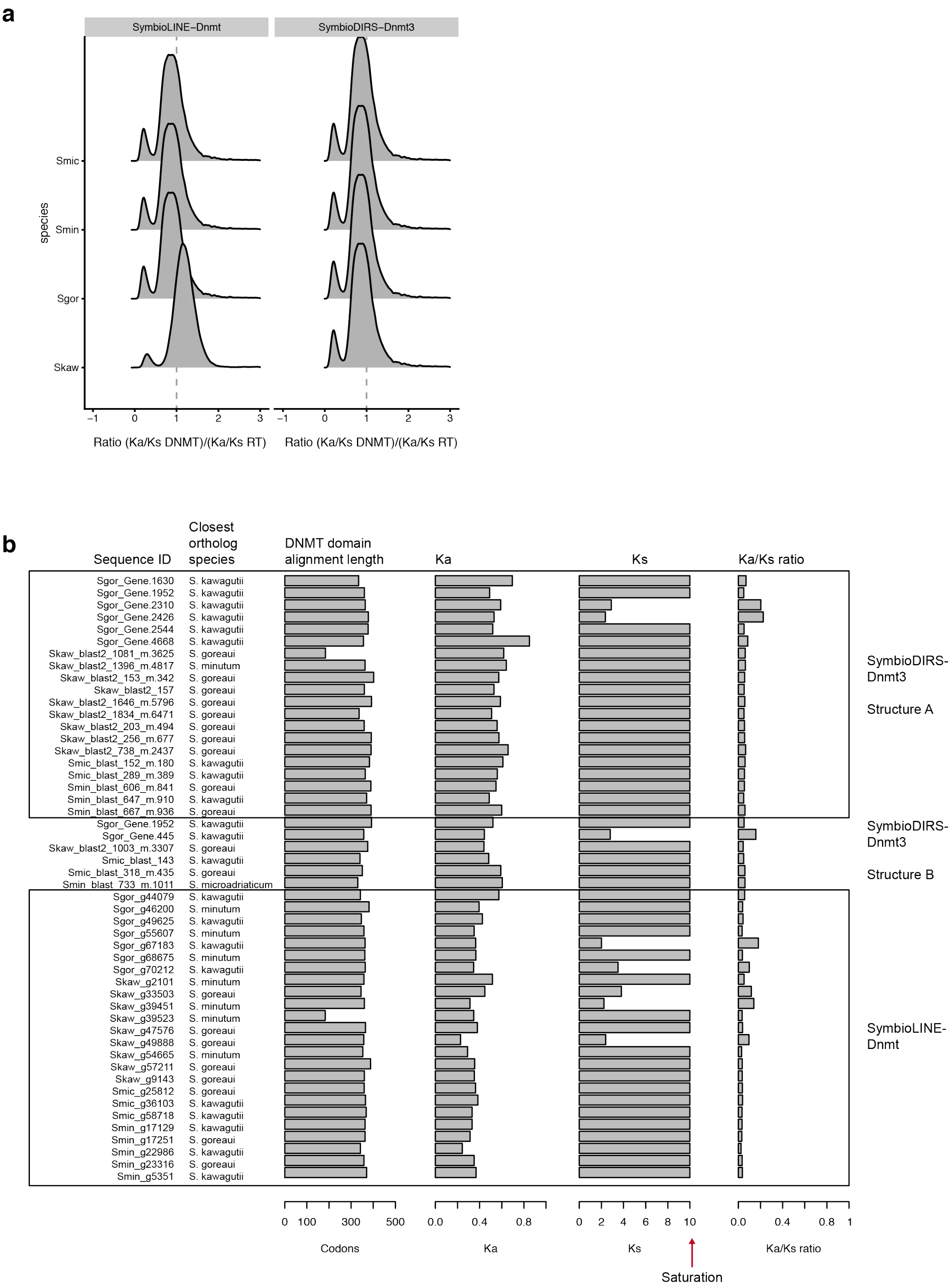

Supplementary Figure 9. Retrotransposon DNMT show evidence of purifying selection across different species. (a) Distribution of ratios of Ka/Ks between DNMT and reverse

transcriptase domains as shown in Supplementary Fig. 8. In most cases, 1 to 1 comparisons show similar levels of purifying selection (ratio ~1) in both domains. **(b)** Barplots showing the pair-wise codon alignments between DNMT belonging to SymbioDIRS-Dnmt3 and SymbioLINE-Dnmt from 4 *Symbiodinium* species. Ks values close to 10 represent saturated substitution rates. Neutral selection is estimated as Ka/Ks ~ 1. Abbreviations: Skaw (*S. kawagutii*), Smin (*S. minutum*), Sgor (*S. goreaui*), Smic (*S. microadriaticum*).

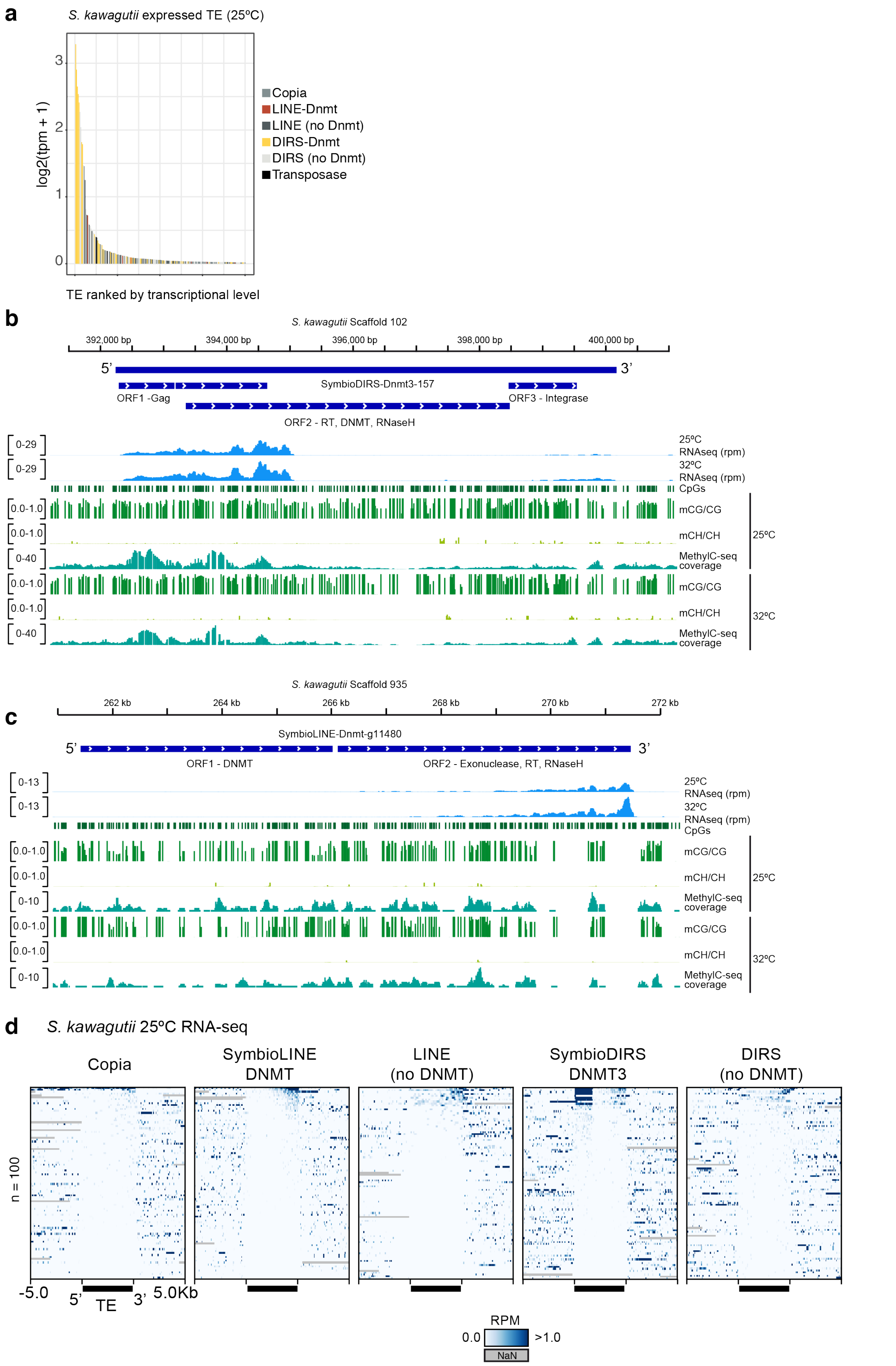
a
S. kawagutii expressed TE (25ºC)
log2(tpm + 1)
3
2
1
0
TE ranked by transcriptional level
Copia
LINE-Dnmt
LINE (no Dnmt)
DIRS-Dnmt
DIRS (no Dnmt)
Transposase
b
S. kawagutii Scaffold 102
392,000 bp
394,000 bp
396,000 bp
398,000 bp
400,000 bp
5'
3'
SymbioDIRS-Dnmt3-157
ORF1 -Gag
ORF3 - Integrase
ORF2 - RT, DNMT, RNaseH
0-29
25ºC RNAseq (rpm)
0-29
32ºC RNAseq (rpm)
CpGs
0.0-1.0
mCG/CG
0.0-1.0
mCH/CH
0-40
MethylC-seq coverage
25ºC
0.0-1.0
mCG/CG
0.0-1.0
mCH/CH
0-40
MethylC-seq coverage
32ºC
c
S. kawagutii Scaffold 935
262 kb
264 kb
266 kb
268 kb
270 kb
272 kb
SymbioLINE-Dnmt-g11480
5'
3'
ORF1 - DNMT
ORF2 - Exonuclease, RT, RNaseH
0-13
25ºC RNAseq (rpm)
0-13
32ºC RNAseq (rpm)
CpGs
0.0-1.0
mCG/CG
0.0-1.0
mCH/CH
0-10
MethylC-seq coverage
25ºC
0.0-1.0
mCG/CG
0.0-1.0
mCH/CH
0-10
MethylC-seq coverage
32ºC
d
S. kawagutii 25ºC RNA-seq
Copia
SymbioLINE DNMT
LINE (no DNMT)
SymbioDIRS DNMT3
DIRS (no DNMT)
n = 100
-5.0
5'
TE
3'
5.0Kb
RPM
0.0
>1.0
NaN

Supplementary Figure 10. Transcriptional and epigenetic profiles of SymbioDIRS-Dnmt3 and SymbioLINE-Dnmt. (**a**) Transposable elements in *S. kawagutii* ranked by transcriptional level as TPM (Transcripts Per Million). (**b**) Genome browser display of an expressed *S. kawagutii* SymbioDIRS-Dnmt3, showing RNA-seq and MethylC-seq tracks for two distinct temperature samples. Temperature conditions do not affect methylation or transcriptional levels. Transcript abundance appears to be higher in both edges of the retrotransposon, mostly not covering *Pol* ORF. Methylation in the CH context is slightly accumulated in the terminal part of the retrotransposon, which might indicate it is in the process of being targeted by CH methylation. (**c**) Genome browser display of an expressed *S. kawagutii* SymbioLINE-Dnmt. Temperature does not appear to affect the methylation or the transcript abundance levels. Here transcripts are biased towards the 3' end of the copy, not covering DNMT-coding ORF1. (**d**) Heatmaps showing transcriptional levels on transposable elements classified by type in *S. kawagutii*. Grey indicates missing values due to lack of coverage (end of scaffolds). Transposable elements are sorted according to expression level and only the top 100 insertions are plotted.

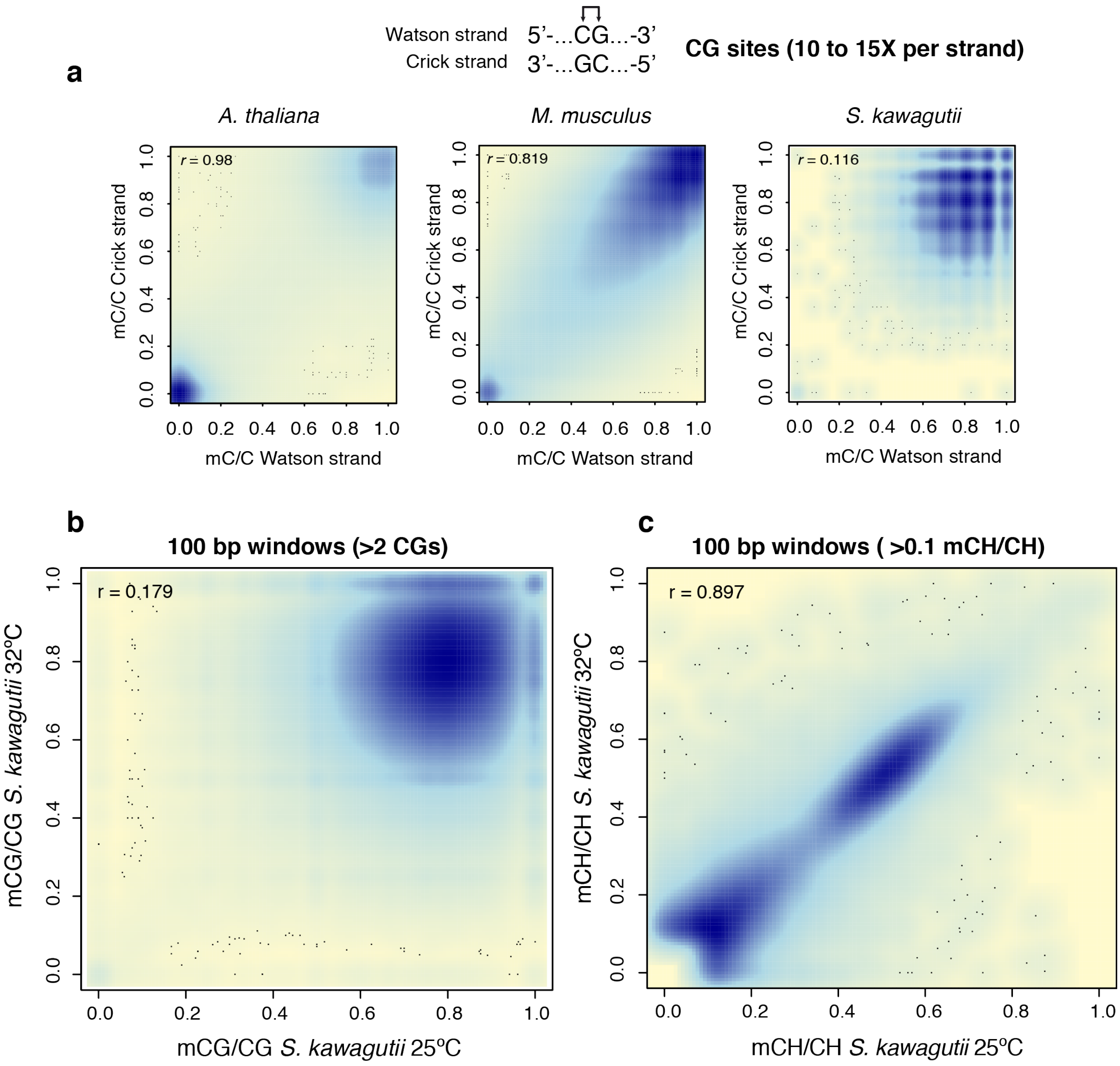

Supplementary Figure 11. Cell heterogeneity of CG methylation in *Symbiodinium*. (**a**) Symmetric methylation in CGs is not well maintained in *S. kawagutii* compared to mouse or *Arabidopsis thaliana*. Low correlation might not be due to strict asymmetry in a single genome, but to diversity of methylation states in single-cell genomes. Coverage per strand has been limited to 10-15 as it captures most CG sites >10x in each sample and allows to compare similarly covered positions across species. (**b**) Comparison of methylation levels in CGs in genome windows of 100 bp between two samples of *S. kawagutii* cultured at different temperatures. Even though the distribution of methylation levels being high in both samples, the correlation is low. (**c**) Comparison of methylation levels in CH context in genome

windows of 100 bp between the same two samples. Windows with CH methylation under 0.1 represent most of the genome, and have been excluded for representation purposes. In stark contrast to methylation in the CG context, CH methylation is well conserved between samples. Both CH and CG windows were filtered for the same coverage (mean C coverage > 4x). CG windows required ≥2 CG sites to be shown as most windows in the genome lacked CG sites.

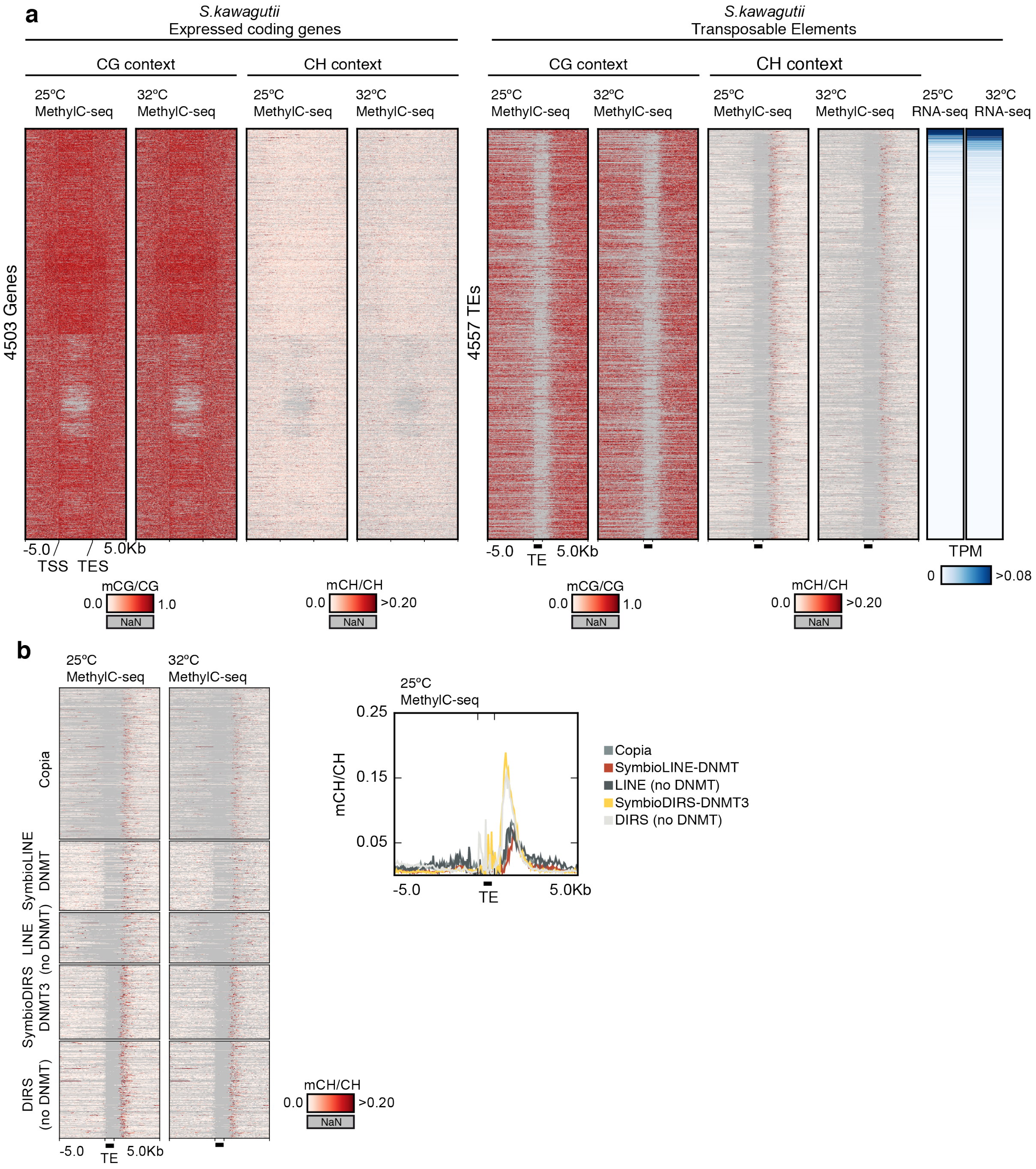

Supplementary Figure 12. CG methylation is not linked to gene expression and CH methylation is enriched in transposable elements in *Symbiodinium*. (**a**) Heatmaps showing methylation levels in the CG and CH contexts for both samples of *S. kawagutii*. Grey indicates missing values due to lack of coverage or missing CGs / CHs in that genomic window. Transposable elements are sorted according to expression level, shown in the right hand side. (**b**) Heatmap and profile of CH context methylation levels on transposable

elements classified by type. DIRS retrotransposons are the most highly methylated regardless of whether they encode a DNMT or not.

a

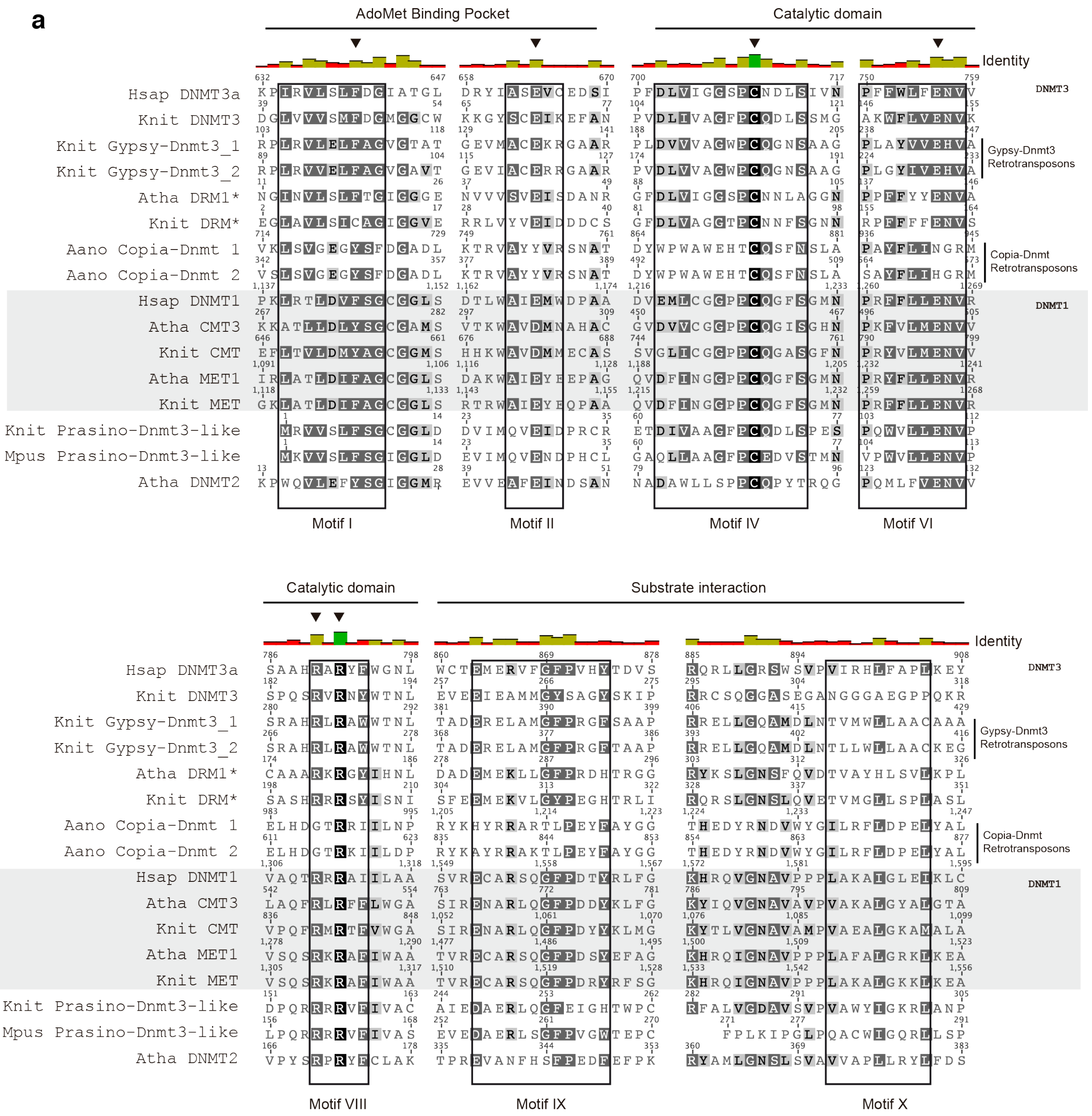

b DNMT domain conservation across KnitGypsy-Dnmt3 copies

Ka/Ks ratio	kfl00004_1	kfl01062_93	kfl01482_133	kfl00111_10
kfl01062_93	0.06454109			
kfl01482_133	0.12700829	0.12544579		
kfl00111_10	0.12559841	0.13554846	0.08920043	
kfl01378_123	0.06069912	0.06130426	0.06142791	0.06352308

Supplementary Figure 13. Sequence conservation of *Klebsormidium* retrotransposon associated DNMTs. (**a**) Multiple sequence alignment showing representative sequences of the distinct DNMTs encoded in *K. nitens* compared to representatives of functionally characterized DNMT classes. Furthermore, *Aureococcus anophagefferens* Copia retrotransposons with a putative DNMT domain are also included. All the diagnostic

functional motifs[1,2] except Motif III are well conserved in most DNMTs. Motif X is not well conserved in *K. nitens* DNMT3 members. In contrast, *A. anophagefferens* Copia retrotransposons show many amino acid changes in the key conserved positions compared to the rest of the eukaryotic DNMTs. The functional amino acids are highlighted with a black triangle. Abbreviations: Hsap (*Homo sapiens*), Atha (*Arabidopsis thaliana*), Mpus (*Micromonas pusilla*), Kfla (*Klebsormidium nitens*), Aano (A. *anophagefferens*). (**b**) Comparison of Ka/Ks values among different copies of KnitGypsy-Dnmt3 DNMT domains.

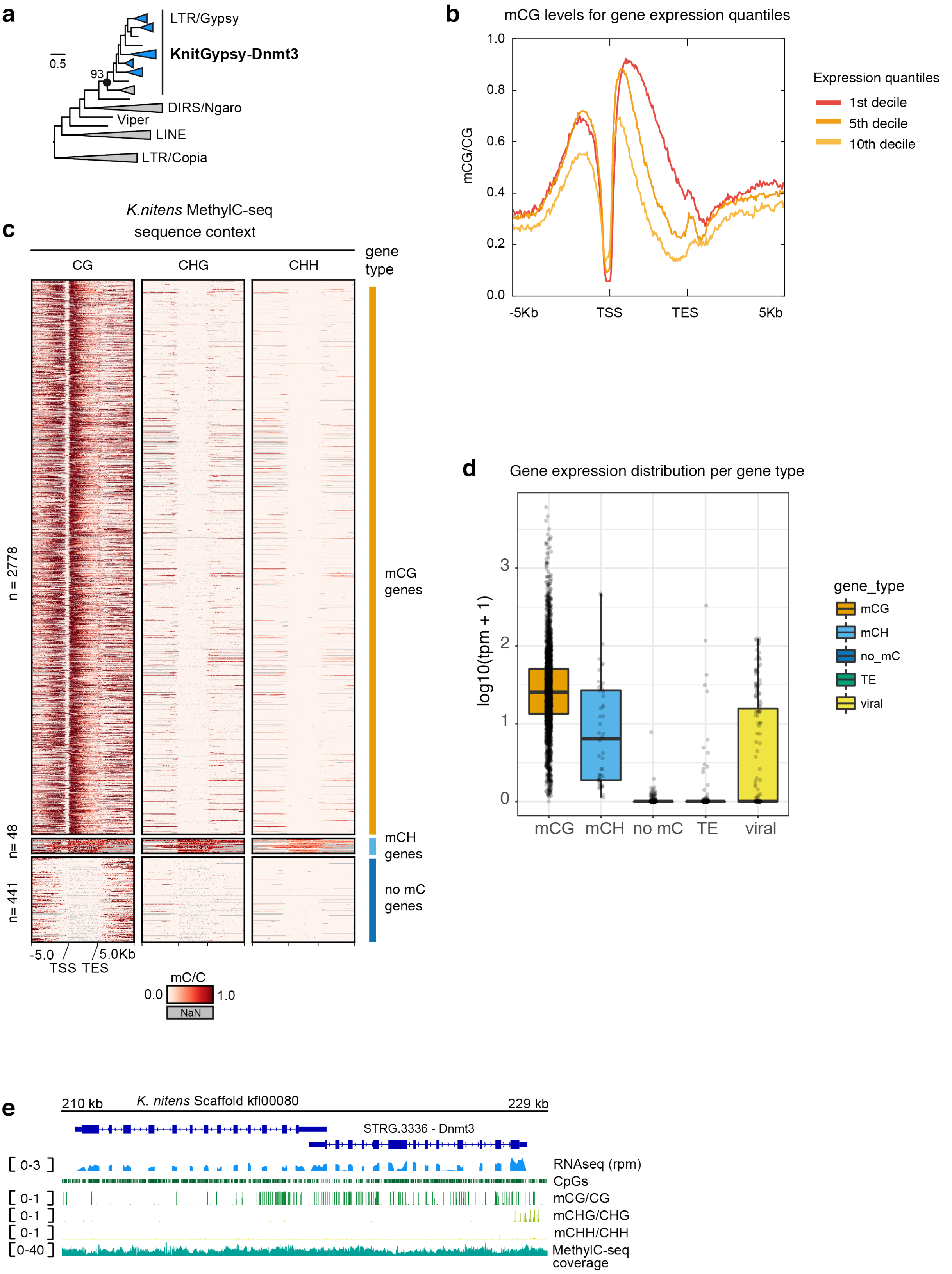
a
LTR/Gypsy
KnitGypsy-Dnmt3
0.5
93
DIRS/Ngaro
Viper
LINE
LTR/Copia
b
mCG levels for gene expression quantiles
mCG/CG
Expression quantiles
1st decile
5th decile
10th decile
-5Kb
TSS
TES
5Kb
c
K.nitens MethylC-seq
sequence context
CG
CHG
CHH
gene type
mCG genes
mCH genes
no mC genes
n = 2778
n= 48
n= 441
-5.0
5.0Kb
TSS TES
mC/C
0.0
1.0
NaN
d
Gene expression distribution per gene type
log10(tpm + 1)
mCG
mCH
no mC
TE
viral
gene_type
mCG
mCH
no_mC
TE
viral
e
210 kb
K. nitens Scaffold kfl00080
229 kb
STRG.3336 - Dnmt3
[0-3]
RNAseq (rpm)
CpGs
[0-1]
mCG/CG
[0-1]
mCHG/CHG
[0-1]
mCHH/CHH
[0-40]
MethylC-seq coverage

Supplementary Figure 14. Sequence context specific methylation and gene transcription in *K. nitens*. (**a**) Maximum likelihood phylogenetic tree of the reverse transcriptase domain showing the affiliation to Gypsy retrotransposon superfamily of KnitGypsy-Dnmt3 sequences. (**b**) Profile of CG methylation levels on gene bodies subdivided by decile of expression. Highly expressed genes show higher gene body methylation levels than lowly expressed genes. (**c**) Heatmaps showing methylation levels in CG, CHH and CHG contexts in a subset of filtered *K. nitens* genes (not including transposable elements or genes of viral origin). The filtering criteria for expressed genes required having annotated UTRs present or encoding conserved protein coding domains for silent genes. Clustering by k-means reveals that there are three types of genes according to the specific methylation context combination on their gene bodies. (**d**) Expression level distribution per gene type, classified as for panel (**c**) heatmap and including transposable elements and viral proteins. A highly reduced subset of transcribed genes are in regions enriched for CG, CHH and CHG methylation, an exception compared to all silent transposable elements or viral proteins found in those hypermethylated regions. Boxplot centre lines are medians, box limits are quartiles 1 (Q1) and 3 (Q3), whiskers are 1.5 x interquartile range (IQR) and points are outliers. (**e**) Genome browser display of the expressed multi-exonic Dnmt3 in *K. nitens*, showing tracks for RNA-seq and MethylC-seq, with methylation in the CG, CHH and CHG sequence contexts shown separately.

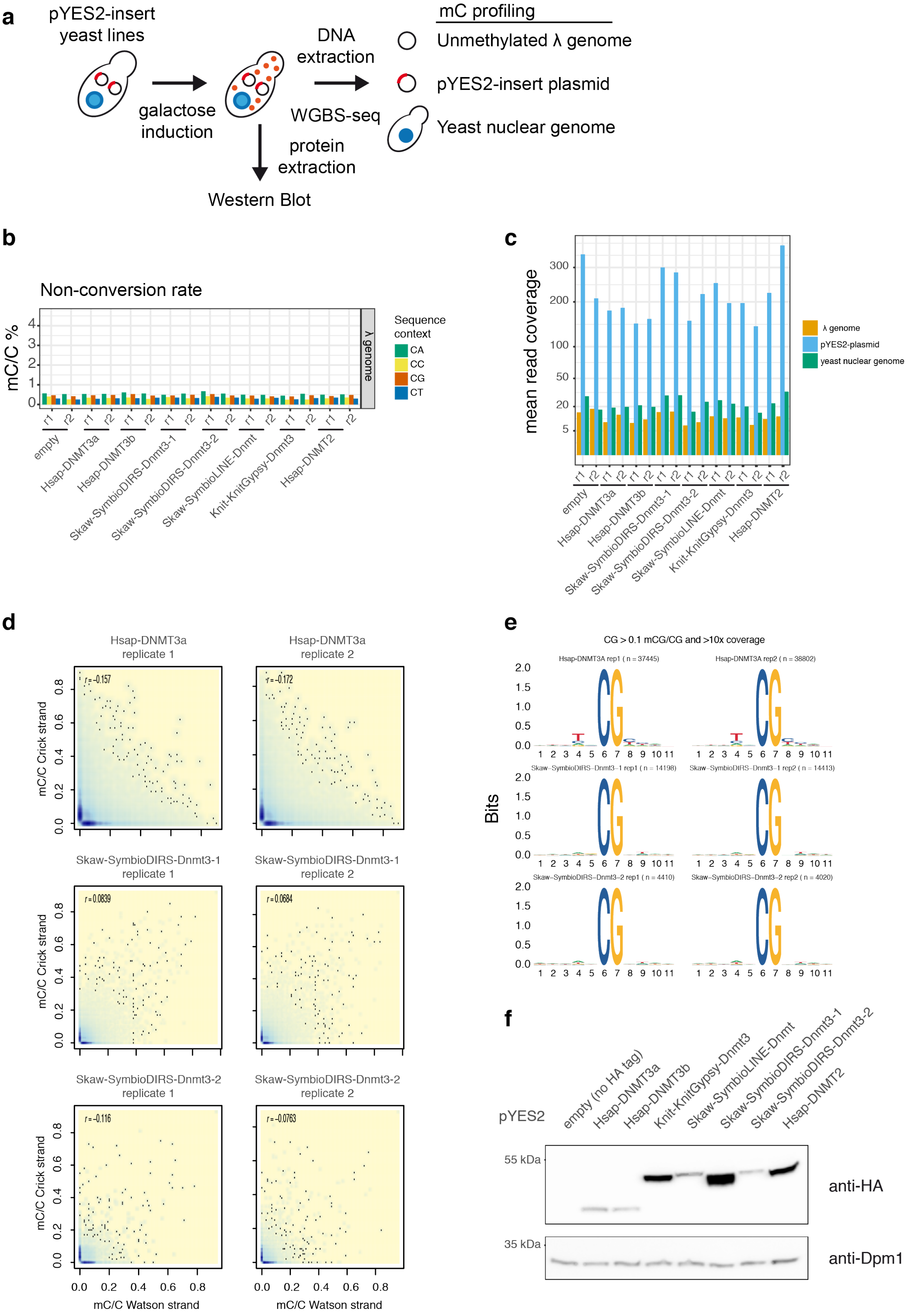
a
pYES2-insert yeast lines
galactose induction
DNA extraction
WGBS-seq
protein extraction
Western Blot
mC profiling
Unmethylated λ genome
pYES2-insert plasmid
Yeast nuclear genome
b
Non-conversion rate
mC/C %
λ genome
Sequence context
CA
CC
CG
CT
empty
Hsap-DNMT3a
Hsap-DNMT3b
Skaw-SymbioDIRS-Dnmt3-1
Skaw-SymbioDIRS-Dnmt3-2
Skaw-SymbioLINE-Dnmt
Knit-KnitGypsy-Dnmt3
Hsap-DNMT2
c
mean read coverage
λ genome
pYES2-plasmid
yeast nuclear genome
d
Hsap-DNMT3a replicate 1
Hsap-DNMT3a replicate 2
Skaw-SymbioDIRS-Dnmt3-1 replicate 1
Skaw-SymbioDIRS-Dnmt3-1 replicate 2
Skaw-SymbioDIRS-Dnmt3-2 replicate 1
Skaw-SymbioDIRS-Dnmt3-2 replicate 2
mC/C Crick strand
mC/C Watson strand
r = -0.157
r = -0.172
r = 0.0839
r = 0.0684
r = -0.116
r = -0.0763
e
CG > 0.1 mCG/CG and >10x coverage
Hsap-DNMT3A rep1 (n = 37445)
Hsap-DNMT3A rep2 (n = 38802)
Skaw–SymbioDIRS–Dnmt3–1 rep1 (n = 14198)
Skaw–SymbioDIRS–Dnmt3–1 rep2 (n = 14413)
Skaw–SymbioDIRS–Dnmt3–2 rep1 (n = 4410)
Skaw–SymbioDIRS–Dnmt3–2 rep2 (n = 4020)
Bits
f
pYES2
empty (no HA tag)
Hsap-DNMT3a
Hsap-DNMT3b
Knit-KnitGypsy-Dnmt3
Skaw-SymbioLINE-Dnmt
Skaw-SymbioDIRS-Dnmt3-1
Skaw-SymbioDIRS-Dnmt3-2
Hsap-DNMT2
55 kDa
35 kDa
anti-HA
anti-Dpm1

Supplementary Figure 15. Experimental methylation induction in *S. cerevisiae.* (**a**) Scheme of the experimental design followed. pYES2-insert transformed yeast cells grown in -URA medium to maintain the pYES2 plasmids. (**b**) Unmethylated spike-in lambda genome methylation as a measure for non-conversion rate in each experiment. (**c**) Mean read coverage per genomic compartment for each experiment. pYES2-insert plasmids are multi-copy in yeast as shown by extensive coverage compared to native yeast genome. Abbreviations: Hsap (*Homo sapiens*), Skaw (*S. kawagutii*), Kfla (*Klebsormidium nitens*). (**d**) Comparison of watson and crick strands methylation levels for sites where mC/C is above 0. None of the samples show evidence for symmetric methylation. (**e**) Sequence logo of the methylation sites where mC/C > 0.1 and coverage is above 10 for the three samples methylation induction was observed. (**f**) Western blot of whole cell extracts of yeast cells expressing the indicated constructs and grown for 24h at 30°C in the presence of galactose to induce expression of DNMTs. anti-HA antibodies (upper panel) were used to detect tested DNMTs and Dpm1 was used as a loading control (lower panel).

Supplementary References

1. Jurkowska, R. Z., Jurkowski, T. P. & Jeltsch, A. Structure and Function of Mammalian DNA Methyltransferases. *Chembiochem* **12,** 206–222 (2011).
2. Gowher, H. *et al.* Mutational analysis of the catalytic domain of the murine Dnmt3a DNA-(cytosine C5)-methyltransferase. *J. Mol. Biol.* **357,** 928–941 (2006).
3. Huff, J. T. & Zilberman, D. Dnmt1-Independent CG Methylation Contributes to Nucleosome Positioning in Diverse Eukaryotes. *Cell* **156,** 1286–1297 (2014).
