# Supplementary material for: Recurrent acquisition of cytosine methyltransferases into eukaryotic retrotransposons
Source: Nat Commun. 2018 Apr 9;9:1341. doi: 10.1038/s41467-018-03724-9 (PMC5890265; doi:10.1038/s41467-018-03724-9)
Supplement: Supplementary file 3 — Description of Additional Supplementary [file 41467_2018_3724_MOESM3_ESM.pdf]

Description of Additional Supplementary Files

Supplementary Data 1.

List of genomes surveyed in the study. Excel file including all phylogenetic affiliations of the genomes used, their complete species names, abbreviation codes for each species, and number of CMT and MBD encoding genes in each genome.
